# Preparation of Acyclovir-Containing Solid Foam by Ultrasonic Batch Technology

**DOI:** 10.3390/pharmaceutics13101571

**Published:** 2021-09-27

**Authors:** Ádám Haimhoffer, Ferenc Fenyvesi, István Lekli, Mónika Béresová, István Bak, Máté Czagány, Gábor Vasvári, Ildikó Bácskay, Judit Tóth, István Budai

**Affiliations:** 1 Department of Pharmaceutical Technology, Faculty of Pharmacy, University of Debrecen, Nagyerdei St. 98, H-4032 Debrecen, Hungary; haimhoffer.adam@pharm.unideb.hu (Á.H.); fenyvesi.ferenc@pharm.unideb.hu (F.F.); vasvari.gabor@pharm.unideb.hu (G.V.); bacskay.ildiko@pharm.unideb.hu (I.B.); 2 Doctoral School of Pharmaceutical Sciences, University of Debrecen, H-4032 Debrecen, Hungary; 3 Institute of Healthcare Industry, University of Debrecen, Nagyerdei St. 98, H-4032 Debrecen, Hungary; 4 Department of Pharmacology, Faculty of Pharmacy, University of Debrecen, Nagyerdei St. 98, H-4032 Debrecen, Hungary; lekli.istvan@pharm.unideb.hu (I.L.); bak.istvan@pharm.unideb.hu (I.B.); 5 Department of Medical Imaging, University of Debrecen, Nagyerdei Krt. 94, H-4032 Debrecen, Hungary; beres.monika@med.unideb.hu; 6 Institute of Physical Metallurgy, Metal Forming and Nanotechnology, University of Miskolc, H-3515 Miskolc-Egyetemváros, Hungary; czmatthews92@gmail.com; 7 Department of Laboratory Medicine, Faculty of Medicine, University of Debrecen, Nagyerdei Krt. 98, H-4032 Debrecen, Hungary; tj0226@med.unideb.hu; 8 Faculty of Engineering, University of Debrecen, Ótemető Str. 2-4, H-4028 Debrecen, Hungary

**Keywords:** ultrasonic, gastroretentive, acyclovir, foam structure

## Abstract

In recent years, the application of solid foams has become widespread. Solid foams are not only used in the aerospace field but also in everyday life. Although foams are promising dosage forms in the pharmaceutical industry, their usage is not prevalent due to decreased stability of the solid foam structure. These special dosage forms can result in increased bioavailability of drugs. Low-density floating formulations can also increase the gastric residence time of drugs; therefore, drug release will be sustained. Our aim was to produce a stable floating formula by foaming. Matrix components, PEG 4000 and stearic acid type 50, were selected with the criteria of low gastric irritation, a melting range below 70 °C, and well-known use in oral drug formulations. This matrix was melted at 54 °C in order to produce a dispersion of active substance and was foamed by different gases at atmospheric pressure using an ultrasonic homogenizer. The density of the molded solid foam was studied by the pycnometer method, and its structure was investigated by SEM and micro-CT. The prolonged drug release and mucoadhesive properties were proved in a pH 1.2 buffer. According to our experiments, a stable foam could be produced by rapid homogenization (less than 1 min) without any surfactant material.

## 1. Introduction

Oral drug delivery systems are the most common dosage forms. They have various advantages, such as good compliance and low costs for storage and transport, and various forms can be manufactured [1]. The physiological variability of the gastrointestinal tract (GI) creates serious challenges for the development of oral drug delivery system formulations. The pH varies in different parts of the digestive tract, and motility depends on meals, as well as enzyme activity [2,3]. Several drugs have poor oral bioavailability due to low or incomplete absorption in the GI [4]. The bioavailability of active ingredients that have an absorption window on the upper part of the GI can be enhanced by formulating gastroretentive dosage forms [5,6]. One of the most important challenges today is antibiotic resistance and protection against various viruses such as COVID-19 [7]. Due to the better bioavailability by gastroretention, more successful therapy and faster remission can be achieved for some diseases and infections. In the case of many antiviral agents, such as zidovudine [8], acyclovir [9,10], or lamivudine [11], better efficacy has been described with gastroretention, and some of these are also available as registered products [12]. Several technologies are currently available to succeed gastric retention [2,4]. Mucoadhesive formulations contain adhesive biopolymers that adhere to the mucosa of the stomach’s inner wall and release an active pharmaceutical ingredient (API) [13,14,15,16]. They usually contain a mucoadhesive polymer such as alginate, chitosan, or even polyethylene glycol [17,18]. In the case of expanding devices, the size increases up to 1.2 cm, inhibiting the transit of the sample to the colon [19]. There are countless possibilities to develop low-density drug carriers [10,20,21] that are able to float on the top of gastric fluid and remain in the stomach until total erosion.

To date, melt-based formulations have been promising new technologies to produce low-density gastroretentive drug delivery systems [21,22,23]. Previously, we developed a novel technology to foam hot and molten dispersions at atmospheric pressure with a rotor–stator homogenizer that is suitable for exploiting the advantages of low-density forms. Furthermore, the product is a non-gas-generating system that is able to float immediately, and the ability of gastric retention was proved in vivo [22,23].

Significant results have been obtained by ultrasonic foaming of metal melt in the metal industry, and based on the results, it can be said that the method is suitable for generating high-porosity solid products from melts [24,25]. The use of ultrasound has long been widespread in healthcare, but to date, it has not been used to produce foam gastroretentive dosage forms.

Our aim was to upgrade our previously published technology with an ultrasonic homogenizer that provides a method for medical use. In this study, the prototype of a novel apparatus is presented: a batch technology to foam melt suspension with different types of gases. Beyond determination of the key parameters, we confirmed that our formulations are low-density, high-porosity products. We applied acyclovir as a model API to prepare and test the foams suitable for gastroretention and possibly provide a new opportunity for the formulations used in the treatment of COVID-19 [26].

## 2. Materials and Methods

### 2.1. Materials

Polyethylene glycol 4000 (PEG 4000), stearic acid, type 50 (SA), and acyclovir (ACV) were of Ph. Eur. grade and purchased from Molar Chemicals Ltd. (Halásztelek, Hungary). Other reagents were of analytical grade and purchased from Sigma Aldrich Ltd. (Budapest, Hungary).

### 2.2. Methods

#### 2.2.1. Development of the Ultrasonic Foaming Equipment

**Figure 1:**
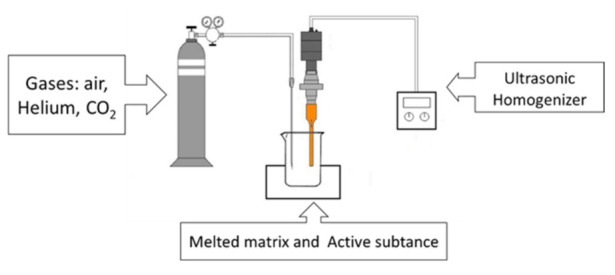
Schematic structure of the ultrasonic foaming device. The three main parts are gas generator, melting vessel, and ultrasonic homogenizer. The apparatus is suitable to create low-density solid foam from melt suspension.

The equipment used in this study is presented in Figure 1. The apparatus can be divided into three main parts: a temperature-controlled vessel with a volume of 50 mL, a Bandelin Sonopuls HD4200 ultrasonic homogenizer with a TS103 sonotrode probe (BANDELIN Electronic GmbH & Co. KG, Berlin, Germany), and a gas injection device that converts the high-pressure gas to atmospheric pressure.

A 50 g amount of melt was foamed by the following method. PEG 4000 and SA were measured and melted in the temperature-controlled vessel at 54 °C. Then, ACV was dispersed in the melted mixture. Foaming was performed by ultrasonic sonication when adding gas into the molten mixture. The procedure was performed for a maximum of 10 s. For further investigations of the sample, the foamed and hot dispersion was molded into a steel mold (V = 1.027 mL, bullet shape [22]) and cooled.

#### 2.2.2. Optimization of Process Parameters

Batch production was optimized using a Box–Behnken experimental design [23]. The independent variables were the gas flow rate (mL/s), foaming time, and sonotrode amplitude (%), and these were considered critical parameters in the production process with an effect on product density (g/cm^3^). These three experimental factors varied in design at 3 levels in 12 runs. The gas flow rate was changed from 0.75 to 3 mL/s, the foaming time from 1.0 to 10.0 s, and sonotrode amplitude was from 10 to 50%. This design was used to investigate the quadratic response surface and to construct a second-order polynomial model using TIBCO Statistica^®^ 13.4 (StatSoft Hungary, Budapest, Hungary).

The 3D response surface plots for density were plotted according to the regression model by keeping one variable at the center level.

#### 2.2.3. Preparation of Floating Acyclovir-Loaded Samples

The acyclovir samples that contained 15% ACV, 75% PEG 4000, and 10% stearic acid were foamed based on the method mentioned above. The independent variables were set to a gas flow rate of 3 mL/s, the sonotrode amplitude was 30%, and the foaming process carried out lasted 8 s. Three compositions were produced by introducing different gases (air, carbon dioxide, helium).

#### 2.2.4. Determination of the Density of Samples

The pycnometer method was used to determine the density of the solid compositions. The total weight was determined by an analytical balance, and the density of each sample was determined with the following formula:

(1) $$\rho_{foam}\;=\;\frac{m_{sample}}{\frac{m_{water}\;-\;\left(m_{water\;+\;sample}\;-\;m_{sample}\right)}{\rho_{water}}}$$

where *m_sample_* is the weight of the ACV-loaded sample, *m_water+sample_* is the weight of the pycnometer, sample, and water, *ρ_water_* is the density of the water at the temperature of water, and *ρ_foam_* is the density of the prepared foam.

#### 2.2.5. Contact Angle Measurement

**Figure 2:**
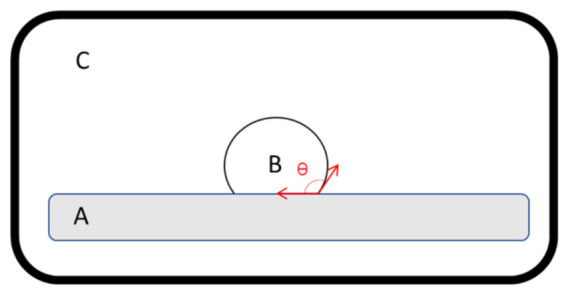
Schematic illustration of the contact angle measurement. (A) High-pressure compressed acyclovir tablet, (B) drop of molten matrix containing PEG 4000 and stearic acid, based on the manufacturing composition, and (C) atmosphere filled with air, helium, or carbon dioxide. The temperature of the apparatus was kept at the melting point of the mixture in an airtight oven. The shape of the molten matrix droplet was imaged, and the contact angle was determined.

Contact angle measurements were performed on a horizontal vacuum tube furnace (Sunplant Ltd., Miskolc, Hungary) equipped with a CCD camera (Figure 2). A drop of melt, without API, was deposited on the surface of the high-pressure compressed acyclovir tablet, while the measuring atmosphere was filled with air, helium, or carbon dioxide. The droplet shape was imaged, and the contact angle was determined using internal software. Measurements were carried out a minimum of five times for each sample, and the results were the average of the measurements.

#### 2.2.6. SEM Analysis and Chemical Element Analysis

A Hitachi Tabletop microscope (TM3030 Plus, Hitachi High-Technologies Corporation, Tokyo, Japan) was used to characterize the solid foams. Samples were split into halves and were attached to a fixture with double-sided adhesive tape containing graphite. Before SEM examination, the gold-sputtered coating was not deposited on the surface of the samples. The measurement required a vacuum and a low accelerating voltage of 5 kV. Chemical element analysis was performed on the fractured surface with a Bruker EDX 70 detector.

#### 2.2.7. Determination of Gas Content

In the case of helium-loaded samples, the gas content was measured after the foaming process. Three pieces of samples were placed into 15 mL vials for sampling gases, and then the samples were heated up to 70 °C until complete melting, after which the trapped gas was released from the melt. The gas composition of the head space was then analyzed by a GC-2010 instrument (Shimadzu, Kyoto, Japan) according to the following method. A 5 mL volume of the headspace gas from each vial was injected into the GC using an MGS-5 gas sampler (Shimadzu, Kyoto, Japan) equipped with a 1 mL calibrated loop in nitrogen gas flow with a speed of 20 cm/s. The injector port temperature was set to 90 °C and was operated in split mode with a split ratio of 50. Isothermal separation of the gases was carried out on a Carboxen 1006 PLOT column (30 m × 0.32 mm × 15 mm) set and maintained at 65 °C. The detector was a thermal conductivity detector with a current of 40 mA. The temperature of the detector was kept at 100 °C.

#### 2.2.8. Microtomography and Size Distribution of Foam Bubbles

The following method was used to determine the solid foam structure. The sample was fixed into the sample holder. A SkyScan 1272 (Bruker, Billerica, MA, USA) compact desktop micro-CT system was used for the measurement. Scanning parameters were the following: image pixel size: 5 microns, matrix size: 1344 × 2016 (rows × columns), source voltage = 50 kV; source current = 200 µA. Flat-field correction and geometrical correction were used. After scanning, the SkyScan NRecon package (version 2.0.4.2) was used to reconstruct the cross-sectional images from the tomography projection images. Post-alignment, beam-hardening correction, ring artifact correction, and smoothing were performed. The output formats were DICOM and BPM images.

In 2D/3D analysis, we used CTAn software (version 1.18.8.0). Based on the density analysis, Thresholding, ROI shrink-wrap, Reload, and 2D and 3D Analysis plugins were used. The gray threshold values of air bubbles were between 0 and 40, and with ROI shrink-wrap, we eliminated the background before analysis. The 3D visualization can be obtained in CTVox software (version 3.3.0) with color coding.

#### 2.2.9. Dissolution Test

Dissolution tests were performed in 900 mL of hydrochloric acid media (pH = 1.2) without pepsin. An Erweka DT 800 dissolution tester and the rotating paddle method were used with a rotation speed of 75 rpm at a temperature of 37 °C. Samples of 3 mL were removed after 5 min, 15 min, and 30 min and then 1, 2, 3, 4, 5, 6, 7, 8, and 10 h. The samples were first filtered through a PES membrane syringe filter (0.22 μm) and then diluted with dissolution medium. The amount of ACV released was determined with a UV/VIS spectrophotometer (Shimadzu UV-1900) at 256 nm. Three random samples from different bulks were selected for the test. Flotation and erosion were evaluated visually and by micro-CT.

#### 2.2.10. Mathematical Analysis of the Drug Release Profiles

Dissolution data were fitted to zero-order, first-order, and Korsmeyer–Peppas models in Microsoft Excel (Table 1).

#### 2.2.11. Floating Strength Determination

**Figure 3:**
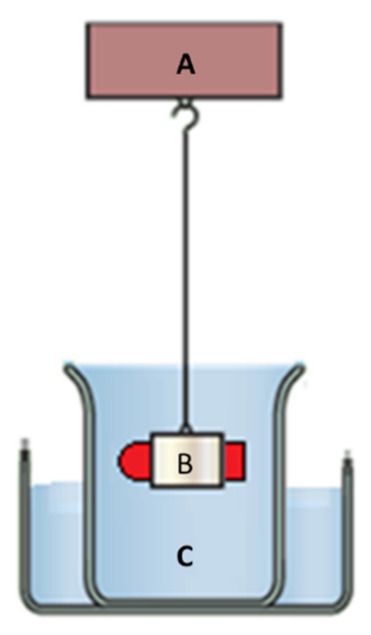
Buoyancy measurements. The net holder (B) is attached to a very sensitive balance (A) while immersing in the release medium (C) along with the samples. Since the net holder is directly mounted to the tensiometer, the rising force can be calculated directly from the weight changes of the net.

Based on the work of Simons and Wagner [21], we built an apparatus capable of detecting the buoyancy force of a sample, as shown in Figure 3. A net holder was directly mounted to the tensiometer (Attension). Thus, the rising force was calculated directly from the weight changes of the net. Measurements were performed in 500 mL of pH 1.2 buffer, kept at 37 °C, and stirred continuously to create comparable conditions to those of in vitro release studies.

#### 2.2.12. Ex Vivo Mucoadhesion Studies

Male Sprague–Dawley (SD) rats were purchased from Charles Rivers (Germany). Animals were nutrified with standard rodent chow ad libitum with free access to water and kept at an ambient temperature of 25 ± 2 °C, with a 12 h light–dark cycle. All animals were treated according to the “Principles of Laboratory Animal Care” formulated by the National Society for Medical Research and the “Guide for the Care and Use of Laboratory Animals” prepared by the National Academy of Sciences and published by the National Institutes of Health (NIH Publication Number: 86-23, revised in 1996). Handling of the animals was approved by the Institutional Animal Care and Use Committee of the University of Debrecen, Debrecen, Hungary (22 July 2021; HB/15-ÉLB/1601-7/2021).

For harvesting the gastric mucosa, rats were overdosed with ketamine (100 mg/kg i.p.). The abdomen was opened, and the stomach was excised and cut open along the lesser curvature. Stomachs were kept in modified Krebs–Henseleit bicarbonate (KHB) buffer (containing 118 mM NaCl, 5.8 mM KCl, 1.8 mM CaCl_2_, 25 mM NaHCO_3_, 0.36 mM KH_2_PO_4_, 1.2 mM MgSO_4_, and 5.0 mM glucose) solution until further use.

Detachment mucoadhesive force studies were performed according to the theoretical base of the modified surface tensiometer method. The inner side of stomach tissues was outspread and immobilized with pins. The samples were fixed with very thin copper wire on a tensiometer arm. Before measurements, mucosae were wetted with 20.0 µL of pH 1.2 buffer in order to achieve better mucoadhesive performance, as previously published [29].

ACV-loading samples were left on the mucosae surface for 3 min to allow wetting and the creation of mucoadhesive bonds. A glass plate was used as a negative control. Maximal detachment forces from mucosa were recorded and corrected for the mass of the sample, which gave the magnitude of the mucoadhesive force formed in mN.

#### 2.2.13. Statistical Analysis

GraphPad Prism^®^ (version 6.01, GraphPad Software Inc., San Diego, CA, USA) was used for statistical analysis. Unp3aired *t*-tests were performed to compare two groups, and one-way ANOVA and Dunnett’s post hoc test were chosen to compare multiple groups. Differences were considered significant at *p* < 0.05.

## 3. Results

### 3.1. Optimization of Process Parameters

**Figure 4:**
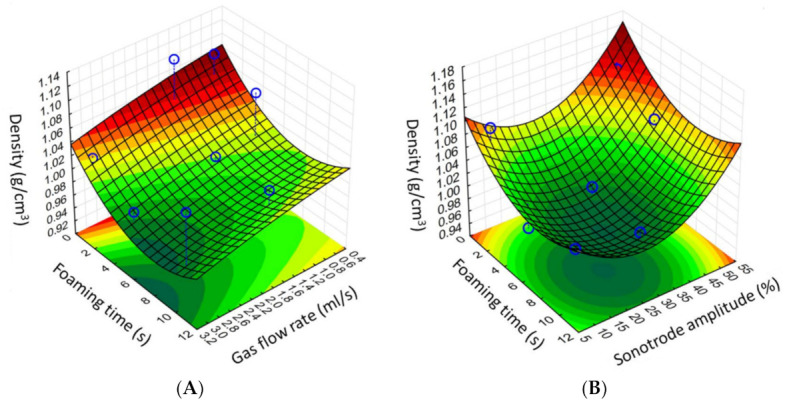
Three-dimensional illustration of density changes during the three-factor experimental design. Sonotrode amplitude (**A**) and gas flow rate (**B**). Independent variables were kept at the center level, while the other two parameters were changed. The dark green range indicates the lowest available density and the ideal foaming parameters.

The effect of gas flow rate (mL/s), foaming time (s), and sonotrode amplitude (%) on the density of samples was investigated. The temperature was set to 54 °C. The results are presented in Figure 4. Increasing the gas flow rate linearly decreased the density of samples in every case. The sonotrode amplitude shows an optimal range between 20 and 30%. In the case of lower values, the rate of foaming was trifling, which could be moderately increased by increasing the foaming time. After the ideal foaming time, the overheating destabilized the foam due to the increased energy transfer. The optimal foaming time was 6 s, which was enough to create a foam structure, avoiding overheated foam aging.

### 3.2. Determination of the Density of Samples

**Figure 5:**
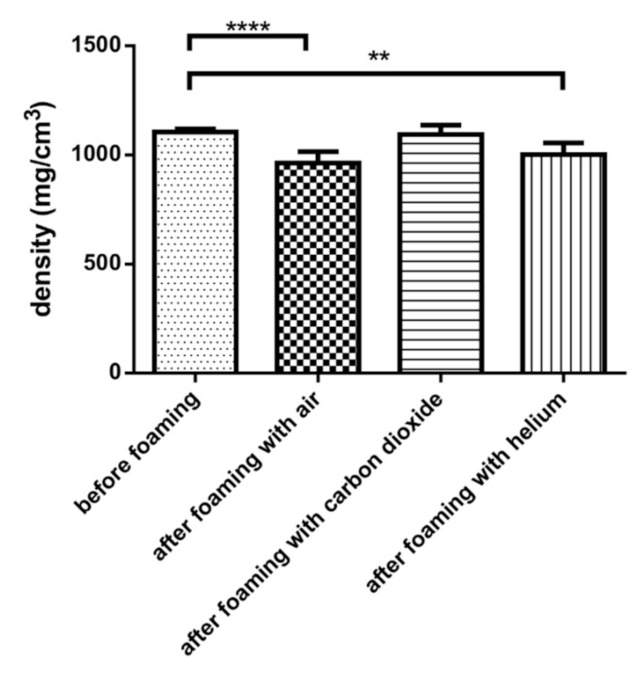
Density of different gas-filled compositions. A significant decrease in density was observed for air and helium but not for carbon dioxide. ** and **** indicate statistically significant differences at *p* < 0.01 and *p* < 0.0001. Data present average values and standard deviations (*n* = 20).

Atmospheric pressure air, carbon dioxide, and helium were used to create foam with the determined optimal parameters. The density of the product is presented in Figure 5. Air was readily available, and CO_2_ was used to create dermatological foams. He is a lighter gas than air and CO_2_ and able to reduce the apparent density of products due to this. In the case of air, we observed a 12% decrease in density, which was sufficient to achieve the buoyancy of the composition from zero minutes. In the case of He, we obtained a significantly lower density than before foaming, and the difference between air and helium was not significant. A negligible decrease in density was observed for CO_2_ that was not significant.

The helium content of the helium-containing samples was measured after formation at the solid structure. The remnant helium content of the samples was only 4.36% of the theoretical maximum content. The theoretical maximum helium content was calculated by the decrease in the samples’ densities. The helium diffuses out the sample during solidification. In the case of carbon dioxide, the residual gas content was not measured due to the absence of a significant decrease in density.

### 3.3. SEM Analysis and Chemical Element Analysis

**Figure 6:**
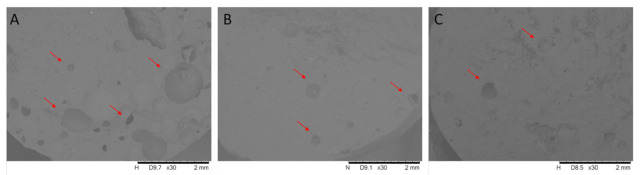
Scanning electron microscopy images of air foaming (**A**), helium (**B**), and carbon dioxide (**C**). The most and largest bubbles are produced in the case of air foaming, while in the case of carbon dioxide foaming, the number of bubbles is negligible. The arrows show cavities/bubbles in the matrix.

As shown in the SEM images (Figure 6), the bubbles can easily be separated from the matrix, and ACV crystals are also detectable. The shape of the cavities is typically spherical or spheroidal in the melt. The bubbles’ independent boundary ceases and merges at a specific point. Scanning electron microscopy confirmed the foam structure that can be produced by ultrasonic mixing. In the case of air foaming, most cavities can be identified in the SEM images. The helium bubbles are smaller, and fewer cavities are present in the matrix, while in the case of carbon dioxide foaming, the number of bubbles is negligible.

**Figure 7:**
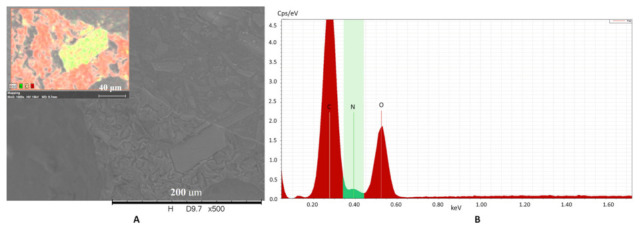
Chemical element analysis shows that API is in solid crystal form (yellowish green), and it is separated from the matrix (red) (**A**). The intensity of the components is represented in the (**B**). (C: carbon; N: nitrogen and O: oxygen element).

Acyclovir crystals are also detectable in the matrix in all compositions. The acyclovir molecule contained five nitrogen atoms per molecule. The matrix did not contain any nitrogen atom, while the number of carbon and oxygen atoms was relatively dominated by API, and the drug crystal and matrix were well separated during elemental analysis. The crystals show a homogenous distribution in the base, and no accumulation is observed at the edges of the bubbles. Element analysis confirmed the crystal form and distribution in the matrix, as shown in Figure 7.

### 3.4. Contact Angle Measurement

To reveal the difference in foaming, a wetting angle measurement was performed in different atmospheres of the foaming gases (Table 2). In the case of air, the homogeneous matrix, the mixture of PEG 4000 and stearic acid, wetted the solid API the best, while the other two gases had a higher contact angle. This could result in worse foaming.

### 3.5. Microtomography and Size Distribution of Foam Bubbles

**Figure 8:**
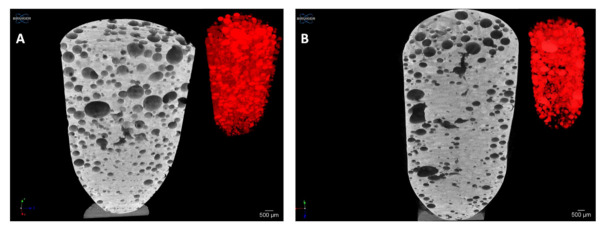
Micro-CT images of solid foam structure (**A**) without and (**B**) with API after foaming with air. In the right corner of the figures, the distributions of bubbles are indicated in red, which prove the homogeneous distribution of the cavities in the sample, and a heterogeneous bubble size distribution can also be observed.

Microtomography images were taken from the samples without API and with API after the foaming process with atmospheric pressure air, shown in Figure 8. The foaming process dispersed gas bubbles into the molten mixture in both cases, as confirmed by the micro-CT images. The distribution of the bubbles was random and homogenous and showed a closed-spheroid cell structure. No accumulation of bubbles was observed at the edges at the bottom or top of the sample. The bubbles were in the 100–1000 μm diameter range. In the case of API composition, the average diameter was 527 µm, while the average diameter of the composition without API was 560 µm. The location of the bubbles is homogeneous, but the bubble size distribution shows heterogeneity.

### 3.6. Dissolution Test

**Figure 9:**
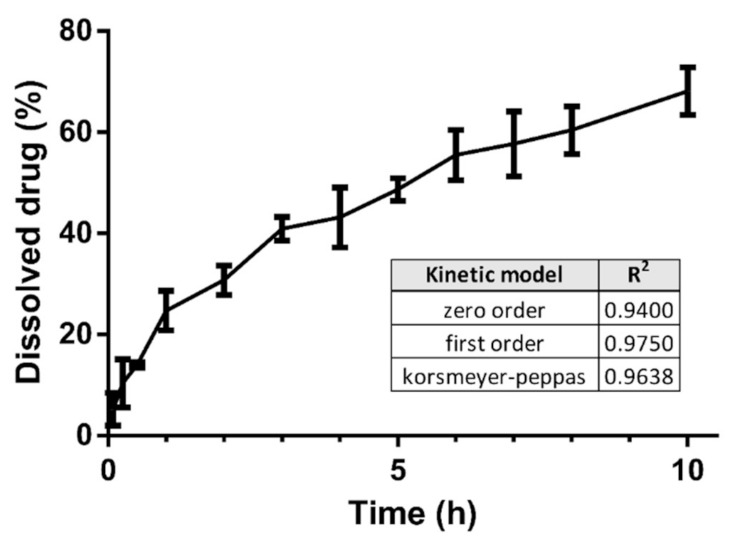
Dissolution profiles of floating air composition. The bars represent mean ± S.D. (*n* = 3). The table summarizes the correlation coefficient for each case of the kinetic model.

Zero floating lag time was proven during the dissolution test. Of the drug, 70% was released up to 10 h. The composition showed continuous buoyancy during the dissolution tests. The drug release profile was analyzed graphically. Zero-order, first-order, and Korsmeyer–Peppas models were used to determine the drug release curve (Figure 9), and determination coefficients were applied to analyze the best fit. The model fitted best with the first-order model, even in the case of zero-order kinetics. We achieved R^2^ = 0.94, which approaches the zero-order drug release.

### 3.7. Floating Force Determination

**Figure 10:**
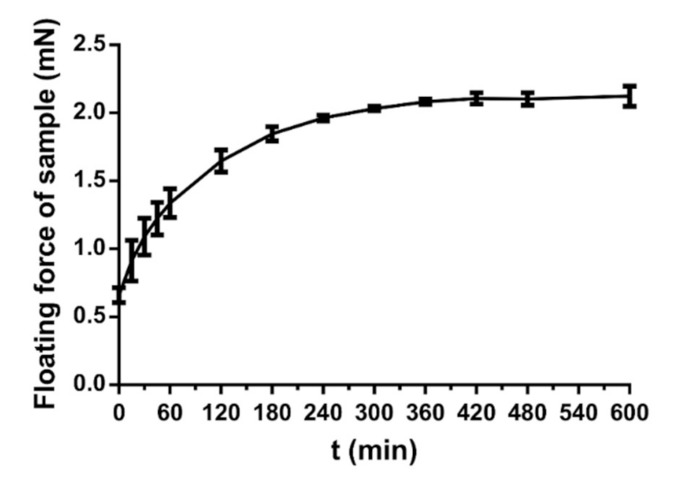
Floating force profile of floating air composition. The bars represent mean ± S.D. (*n* = 3).

During the dissolution study, the buoyancy force of the sample was 0.66 mN at 0 min. In the first four hours, we could see a steep curve profile, as shown in Figure 10. The floating force increased rapidly up to 240 min, where it reached a force of 1.96 mN. Between 240 and 600 min, the floating force increased minimally and took up a plateau section.

**Figure 11:**
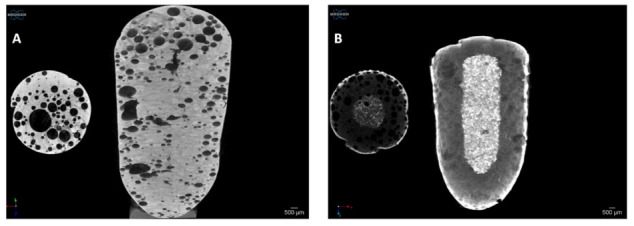
Micro-CT images of solid foam structure (**A**) at zero time and (**B**) 10 h after dissolution test. The solid, unwetted core remained in the center of the specimen’s body after 10 h of dissolution (white area in (**B**)).

Due to dissolution, the water-soluble matrix and the ACV were dissolved from the formulation after 10 h, and the water-insoluble matrix, stearic acid, remained in the original body of the sample, as presented in Figure 11. The average of the percentage of remaining mass of the samples is only 13.3 ± 1.35%.

### 3.8. Ex Vivo Mucoadhesion Studies

**Figure 12:**
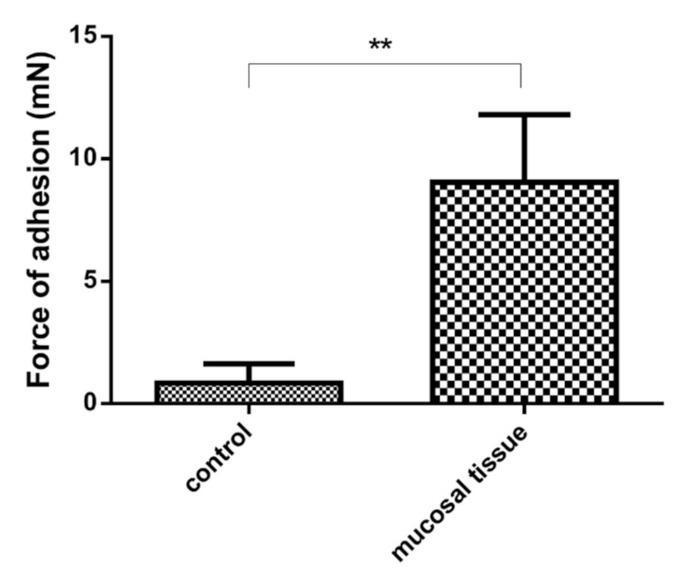
Mucoadhesive force of samples on inner surface of rat’s stomach and control adhesion on glass surface. Significantly higher mucoadhesive force was detected on mucosal tissue than in case of control. ** indicates statistically significant differences at *p* < 0.01.

In the mucoadhesion study, we demonstrated that our product is able to adhere to the inner surface of the stomach and thereby further increase the residence time in the stomach. An average force of 9 mN was required to remove and wash off the gastric surface. Compared to the flat glass surface used as a control, this represents a 9-fold increase in force, which is significantly higher (Figure 12).

## 4. Discussion

Our aim was to upgrade our previously published technology [22,23] with an ultrasonic homogenizer that provides a method for medical use. The ultrasonic homogenizer is suitable to create metal foams [24,25]. The technology is transferable to pharmaceutical science and compatible with numerous polymers [30]. There is a limited number of similar methods in the literature for the preparation of a low-density gastroretentive composition based on melt formation [21,22,23,31,32].

Our in-house foaming apparatus was designed, built, and optimized to foam molten dispersions by ultrasonic energy. The components of the matrix, PEG 4000 and SA type 50, are biocompatible materials that are well described in the literature [33,34] and have sufficiently low melting and freezing points. The foaming device includes three basic units with a container of approximately 100 mL. One of the disadvantages of batch melting technologies is the capacity, but our equipment has a higher capacity compared to our previously described method [22] and may even be suitable for larger-scale production than already published technologies [21,23]. The vessel’s temperature can be kept at a constant value between 30 and 70 °C, which covers a wide range of melting points of the polymers that can be used. The main foaming was performed in the container, in which the gas was dispersed into the molten dispersion with the ultrasonic homogenizer. The foam was poured into metal molds and cooled down to room temperature until complete solidification. During optimization of the process parameters, the ideal production temperature was determined to be 54 °C, but the optimal temperature depended on the composition, as determined previously [23]. We found that by increasing the gas flow rate, the density of samples decreased linearly in every case. Optimal foaming was performed at 30% of the maximum amplitude for 6 s. In cases of lower values, the rate of foaming is trifling, which can be moderately increased by increasing the foaming time. After the ideal foaming time, foam destabilization occurs due to overheating [35,36] as a result of increased energy transfer, which was reflected in the three-dimensional illustration of the three-factorial experimental design. In the case of DoE, the reproducibility of the production was also looked into, and the densities obtained were compared and examined.

Atmospheric pressure air, carbon dioxide, and helium were used to create the foams. In the case of air, we noticed a 12% decrease in density, which was enough to achieve the buoyancy of the composition from zero minutes. This decrease in density was less than in the case of previous results with high agitation or hot-melt extrusion [21,22,23,31,32]. In the case of two pure gases, CO_2_ and He, the foaming was moderate or negligible, which is well reflected in the different contact angle results. The increasing wetting contact angle can lead to poorer foaming [37]. CO_2_ was previously used successfully in hot-melt extrusion to create high-porosity carriers, but in these cases, either CO_2_ released from the NaHCO_3_ or the mixture of pressurized CO_2_ and sodium bicarbonate was used [31,32]. Foams prepared by helium have been described in many cases, but these foams were not solid foams produced by melting technology. In other published studies, highly porous formulations could be achieved by forming 200–300 μm cavities [31,38]. The bubbles in the present composition show a size distribution in the diameter range of 100–1000 μm. The average diameter was 527 µm, which shows larger bubbles and heterogeneous distribution than our results with high agitation [22,23]. One of the disadvantages of the current batch technology is the intermittent pouring and cooling, which indicates the system’s susceptibility to the environment’s different conditions, such as laboratory temperature. Further experiments and developments are necessary to decrease the foaming system’s intra- and interbatch variabilities. A possible method to achieve this is to integrate the presented methodology into a continuous production process. Despite the average densities of the foams, the uniformity of the mass of the molded products is within the pharmacopeia limits, but the bubbles can still merge, causing a heterogeneous size distribution. In terms of the dissolution profile of the air-foamed sample, it releases its active ingredient with prolonged release. The heterogenous bubble’s size distribution does not significantly affect the release kinetics of the drug during the test. Compared to the other acyclovir formulations published earlier, we achieved a longer, nearly constant-release kinetic during 10 h of dissolution [10,32]. In vivo studies show that GR systems can achieve a prolonged plasma drug concentration that increases the bioavailability of ACV [10,39]. Conventional pills (immediate release) and prolonged-release tablets do not show different bioavailabilities; however, slow dosing, over 4 h, of infused acyclovir solution in the duodenum and the sipped solution increased the AUC areas in the human study [10,40]. Interestingly, during dissolution, the buoyancy increases continuously, which is demonstrated by the micro-CT images, as the size of the formulation does not decrease, but the internal soluble matrix dissolves, thus reducing the relative density of the formulation, leaving only a stearic acid backbone due to poor water solubility. The remaining samples are fragile and can thus be easily eliminated from the stomach after dissolution of the drug, as our previous results have already demonstrated in animal experiments [23]. The samples contained a high amount of PEG 4000 that made them suitable for mucoadhesion [18]. Our measurements revealed that twice the weight of the preparation is needed to tear it off the stomach mucosa surface. This property can further increase its residence time in the stomach. This mucoadhesive force is less than that detected in the formulation with one of the most frequently used hydrophilic polymers [13,41]. On the other hand, the PEG content of our formulation may contribute to a prolonged gastric residence time.

The developed method provides an opportunity to achieve rapid individual medication in hospitals when a low-density gastroretentive preparation should be applied. Furthermore, the bioavailability of acyclovir could be increased by delivering the active ingredient more slowly and evenly than in previously published formulations. The technology also further expands the possibility of gastroretentive applications of antiviral ingredients.

## 5. Conclusions

A novel apparatus was designed and built for the foaming of molten dispersions by ultrasonic agitation. The foaming process was optimized by a Box–Behnken experimental design to determine the most effective setup to create solid foams. We developed high-porosity acyclovir samples with air, helium, and carbon dioxide. We applied several methods to characterize the properties of the foam matrix system. SEM images and micro-CT scans confirmed that bubbles form a spherical closed-cell structure, where clusters of interconnecting voids can be found. The zero-order correlation coefficient of the dissolution curve was greater than 0.94. The samples show a mucoadhesive property on rat stomach mucosae surface. This study provides a promising platform for marketed active ingredients with low bioavailability and expands the possibility of gastroretentive applications of antiviral ingredients.

## Figures and Tables

**Table 1:** Mathematical model of drug release profiles.

Model	Equations [27,28]		Graphic
Zero-order	$Q_{t}\;=\;Q_{0}\;+\;k_{0}t$	(2)	The graphic of the drug-dissolved fraction versus time is linear.
First-order	$Q_{t}\;=\;Q_{0}\;\times \;e^{-k_{1}t}$	(3)	The graphic of the decimal logarithm of the released amount of drug versus time is linear.
Korsmeyer–Peppas model	$\frac{Q_{t}}{Q_{\infty }}\;=\;k_{kp}t^{n}\left(\mathrm{up}\;\mathrm{to}\;\frac{Q_{t}}{Q_{\infty }}\;\geq \;0.6\right)$	(4)	The graphic of the released drug versus the square root of time should form a straight line.

where *Q*_0_ is the initial amount of drug; *Q_t_* is the amount of drug remaining at time *t*; *Q_t_/Q_∞_* is the fraction of drug released at time *t*; *k*_0_, *k*_1_, and *k_kp_* are the kinetic constants; and *n* is the release exponent indicative of the drug release mechanism.

**Table 2:** Contact angles in the presence of foaming gases in the three-phase wetting angle measurement.

Type of Gas	Contact Angle (° ± SD)
Air	11.74° ± 2.01
Carbon dioxide	17.89° ± 1.63
Helium	18.66° ± 1.54

## Data Availability

Not applicable.
